# Retinopathy and Uveitis Associated with Sofosbuvir Therapy for Chronic Hepatitis C Infection

**DOI:** 10.7759/cureus.597

**Published:** 2016-05-03

**Authors:** Katrina Chin-Loy, Farah Galaydh, Saad Shaikh

**Affiliations:** 1 Ophthalmology, Howard University College of Medicine; 2 Ophthalmology, Orlando Veterans Affairs Medical Center; 3 Ophthalmology, UCF College of Medicine; 4 Ophthalmology, USF College of Medicine; 5 Ophthalmology, University of Texas Medical Branch at Galveston; 6 Ophthalmology, Florida State University College of Medicine

**Keywords:** uveitis, retinopathy, sofosbuvir, sensorineural hearing loss, rheumatology, essential tremor, toxicity, drug

## Abstract

Purpose: We report a case of retinopathy and uveitis associated with sofosbuvir therapy for hepatitis C infection.

Methods: Case report.

Results: A 57-year-old-male developed ocular inflammation and retinopathy four weeks after the administration of sofosbuvir for a hepatitis C infection. Hearing loss, rheumatologic disease, and essential tremor were also noted. The ophthalmic findings resolved with discontinuation of the drug.

Conclusion:  The authors report a case of sofosbuvir induced retinopathy and uveitis, the first associated with this emerging therapy for hepatitis C. Ophthalmologists and other treating physicians should be aware of the ophthalmic side effects of this drug.

## Introduction

Direct-acting antivirals (DAAs) are a new class of therapeutics that target specific stages of hepatitis C viral (HCV) replication. Sofosbuvir, an oral nucleotide analogue inhibitor of HCV NS5B polymerase, acts as a chain terminator when incorporated into HCV RNA [1]​.  A 12-week course of sofosbuvir, with ribavirin, results in an 86-93% sustained virologic response (SVR) for HCV genotype 2 [1]. We report a case of uveitis and retinopathy associated with sofosbuvir use.

## Case presentation

A 59-year-old Caucasian male with chronic HCV and well-controlled hypertension presented with a four-week history of bilateral red eyes, tearing, blurred vision, photophobia, and decreased hearing. He was also rheumatoid factor (RF)-positive and antinuclear antibody (ANA)-positive, which his rheumatologists felt were secondary to a chronic HCV infection. He had no previous history of retinopathy, uveitis, or other ophthalmic or visual complaints. Eight weeks prior, he was started on a course of oral ribavirin, 600 mg twice daily, and sofosbuvir, 400 mg daily, for treatment-naïve HCV infection. Four weeks later, he reported the aforementioned ocular and auditory complaints and had developed anemia. The ribavirin dose was reduced to 400 mg daily while the sofosbuvir dose remained unchanged. Two weeks later, he noted worsening vision, hearing loss, gait instability with tremor, joint pain, nausea, and fatigue. His ribavirin and sofosbuvir were both discontinued. Audiology evaluation revealed bilateral moderate to severe sensorineural hearing loss requiring hearing aids. New onset essential tremor and non-rheumatologic joint pain were diagnosed by the physiatry and rheumatology services, respectively. Ophthalmic evaluation revealed a vision of 20/40 and 20/50 in his right and left eyes, respectively. Anterior segment exam showed conjunctival injection, scattered, fine keratic precipitates, and 3+ cell and flare bilaterally. Dilated fundus exam revealed bilateral peripapillary cotton-wool spots but was otherwise unremarkable. He was diagnosed with acute anterior non-granulomatous uveitis and suspected drug-associated retinopathy and was started on prednisolone acetate and cyclopentolate ophthalmic drops. A laboratory workup for etiologies of the uveitis was negative, except his positive RF and ANA values. Eleven weeks after cessation of ribavirin and sofosbuvir, his vision was 20/20 and 20/25 with a resolution of the uveitis and cotton-wool spots after tapering off the prednisolone acetate. Lab work at that time revealed that the patient’s HCV was undetectable by polymerase chain reaction (PCR) testing. Informed patient consent was obtained for this patient's treatment.

## Discussion

Although sofosbuvir has been associated with ocular surface changes, no previously reported associations with uveitis or retinopathy exist in the literature [2]. The temporal onset of ophthalmic findings shortly after initiation of sofosbuvir and ribavirin in our patient supports a drug-related etiology. Hepatitis C virus infection-induced autoimmune processes have previously been associated with seroconversion of various indexes of autoimmunity, including RF and ANA [3]. It is not clear in this case if his RF+ and ANA+ status or any underlying inflammatory disease contributed to these findings but is unlikely the primary etiology, given the absence of prior ophthalmic disease. Whether the ribavirin alone was etiologic cannot be excluded but is unlikely, given dose reduction did not prevent the progression of symptoms.

The pathogenesis of sofosbuvir-related retinopathy may share common features with the development of interferon (IFN) retinopathy and hearing loss [4]. Several studies propose that IFN toxicity may be due to an inflammatory autoimmune process or focal small vessel vasculitis [4].As with IFN, ribavirin may have played a synergistic role in our case. Unlike IFN, the newer antivirals in the class of direct antiviral agents (DAAs) are thought to have less systemic side effects and propensity to cause autoreactivity because they directly target HCV RNA. Chronic HCV infection itself has been commonly associated with autoimmunity, perhaps by reducing tolerance to self-antigens [5]. Our patient differed from the classic presentation of IFN retinopathy in that he also demonstrated an anterior segment inflammatory reaction, essential tremor, and joint pain, and this may suggest that other pathogenetic mechanisms may be involved.

## Conclusions

We report here for the first time a sofosbuvir-induced retinopathy and uveitis. Ophthalmologists and HCV-treating physicians should be aware of ophthalmic side effects associated with this emerging therapy for hepatitis C infections. 
