# Correction: Retinopathy and Uveitis Associated with Sofosbuvir Therapy for Chronic Hepatitis C Infection

**DOI:** 10.7759/cureus.c3

**Published:** 2016-07-14

**Authors:** Katrina Chin Loy, Farah Galaydh, Saad Shaikh

**Affiliations:** 1 Ophthalmology, Howard University College of Medicine; 2 Ophthalmology, Orlando Veterans Affairs Medical Center; 3 Ophthalmology, UCF College of Medicine; 4 Ophthalmology, USF College of Medicine; 5 Ophthalmology, University of Texas Medical Branch at Galveston; 6 Ophthalmology, Florida State University College of Medicine

The Results section in the Abstract erroneously reads "57-year-old-male” but should instead read "59-year-old-male” as it is correctly written later in the article.

